# Homologous Recombination Pathway Alternation Predicts Prognosis of Colorectal Cancer With Chemotherapy

**DOI:** 10.3389/fphar.2022.920939

**Published:** 2022-06-06

**Authors:** Yan Lin, Xiaoli Liao, Yumei Zhang, Guobin Wu, Jiazhou Ye, Shanshan Luo, Xinxin He, Min Luo, Mingzhi Xie, Jinyan Zhang, Qian Li, Yu Huang, Sina Liao, Yongqiang Li, Rong Liang

**Affiliations:** ^1^ Department of Medical Oncology, Guangxi Medical University Cancer Hospital, Nanning, China; ^2^ Department of Hepatobiliary Surgery, Guangxi Medical University Cancer Hospital, Nanning, China; ^3^ Department of Gastrointestinal Surgery, Guangxi Medical University Cancer Hospital, Nanning, China

**Keywords:** colorectal cancer, chemotherapy, biomarker, prognosis, homologous recombination

## Abstract

**Background:** Chemotherapy is the basic treatment for colorectal cancer (CRC). However, colorectal cancer cells often develop resistance to chemotherapy drugs, leading to recurrence and poor prognosis. More and more studies have shown that the Homologous recombination (HR) pathway plays an important role in chemotherapy treatment for tumors. However, the relationship between HR pathway, chemotherapy sensitivity, and the prognosis of CRC patients is still unclear.

**Methods:** We collected 35 samples of CRC patients after chemotherapy treatment from Guangxi Medical University Cancer Hospital, then collected mutation data and clinical prognosis data from the group. We also downloaded Mondaca-CRC, TCGA-CRC cohorts for chemotherapy treatment.

**Result:** We found that HR mutant-type (HR-MUT) patients are less likely to experience tumor metastasis after receiving chemotherapy. Additionally, our univariate and multivariate cox regression models showed that HR-MUT can be used as an independent predictor of the prognosis of chemotherapy for CRC patients. The KM curve showed that patients with HR-MUT CRC had significantly prolonged overall survival (OS) time (log-rank *p* = 0.017; hazard ratio (HR) = 0.69). Compared to HR mutant-type (HR-WT), HR-MUT has a significantly lower IC50 value with several chemotherapeutic drugs. Pathway enrichment analysis further revealed that the HR-MUT displayed a significantly lower rate of DNA damage repair ability, tumor growth, metastasis activity, and tumor fatty acid metabolism activity than HR-WT, though its immune response activity was notably higher.

**Conclusion:** These findings indicate that HR-MUT may be a relevant marker for CRC patients receiving chemotherapy, as it is closely related to improving OS time and reducing chemotherapy resistance.

## Introduction

Colorectal cancer, or colon cancer, is one of the most common types of malignant tumors found in the human digestive tract and is a seriously threat to human health. Its incidence rate ranks third amongst that of all malignant tumors worldwide, and its mortality rate ranks second (Miller et al., 2020; Liu et al., 2022a; Liu et al., 2022b). At present, besides surgery, chemotherapy is the main treatment for colon cancer. However, due to the high heterogeneity of colon cancer, the benefits of these treatments for difference patients may vary greatly. Moreover, the occurrence of chemotherapy resistance can result in failure of the final treatment of colon cancer patients (Bian et al., 2016; Fu et al., 2016). Therefore, it is of the utmost importance to investigate the specific mechanism of the occurrence and development of colon cancer and any accompanying chemotherapy resistance in order to develop new therapeutic targets, reverse chemotherapy resistance, and ultimately improve the prognosis of colon cancer patients.

Recent studies have shown that the molecular mechanisms of chemotherapy resistance mainly involve: 1) The reduction of the activation of drug precursors and the concentration of drugs in cells through drug efflux and inactivation; 2) Changes in drug action targets; 3) Disturbance of cell survival and apoptosis; 4) Hypoxia in the tumor microenvironment; 5) Changes in the extracellular mechanism; 5) Cytokines and other growth factors that maintain the activation of tumor survival-related pathways (Li et al., 2017).

Homologous recombination (HR) is a stable and error-free repair process that uses homologous sister chromatids as a template. HR determines the survival and fate of cells (Gonzalez and Stenzinger, 2021; Liu et al., 2021). Still, despite its reliability, the main cause of DNA damage in the S/G2 cell cycle is HR (Lisby and Rothstein, 2015). HR plays an important role in repairing cisplatin adducts, a DNA repair process that plays an important role in the chemotherapy resistance of tumor cells (Zdraveski et al., 2000). For example, HR-deficient Escherichia coli strains are known to show higher sensitivity to chemotherapeutic drugs than Nucleotide excision repair (NER)-deficient Escherichia coli strains (Zdraveski et al., 2000), while homologous recombination defect (HRD) is known to be related to chemotherapy resistance in ovarian cancer patients (Xiao et al., 2017). However, at present, the relationship between the mutation state of the HR pathway and the chemotherapy efficacy, prognosis, and chemotherapy sensitivity of CRC patients is unclear.

In this study, we use data collected from a sample of CRC patients who have been treated with chemotherapy from Guangxi Medical University Cancer Hospital to explore the relationship between the mutation state of HR pathway and the prognosis of the chemotherapy efficacy of CRC patients through curative effect analysis, prognosis analysis, and drug sensitivity analysis. Through this process, we found that HR-MUT CRC patients had significantly prolonged survival time and higher sensitivity to chemotherapy drugs. Therefore, HR-MUT may be a useful predictive marker for CRC patients receiving chemotherapy in future treatments.

## Materials and Methods

### CRC Cohort Collection

This study includes samples of primary CRC patients that underwent colon cancer resection and chemotherapy in Guangxi Medical University Cancer Hospital between 2015 to 2021. The sample patients were divided into patients with tumor metastasis and without tumor metastasis. We obtained the mutation data by targeted sequencing. We also collected the clinical data of the 35 patients, including their level of metastasis, sex, TNM stage, Eastern Cooperative Oncology Group Performance Status (ECOG), age, height, and weight. All participants signed a written informed consent agreement, and the work was approved by the Research Ethics Committee of Guangxi Medical University Cancer Hospital.

To expand our data, we downloaded a CRC cohort for chemotherapy (Mondaca-CRC; https://www.cbioportal.org/study/clinicalData?id=crc_apc_impact_2020) from the cbioportal web tool (Mondaca et al., 2020), as well as the entirety of the exon sequencing data and clinical data (including Tumor mutational burden (TMB), Microsatellite instability (MSI) scores, MSI status, gender, TNM stage, ECOG and age). We downloaded the RNA sequencing data, mutation data, and clinical prognosis data of TCGA-COAD and TCGA-READ from TCGA database (https://portal.gdc.cancer.gov/) using TCGAbiolinks R package (Colaprico et al., 2016). We then combined TCGA-COAD cohort and TCGA-READ cohort into TCGA-CRC cohort, which was used for our subsequent analysis. The TMB of TCGA-CRC was downloaded from the previous literature (Lin et al., 2020). Clinical basic information about Local-CRC, Mondaca-CRC and TCGA-CRC are defined in Supplementary Table S1–S3, respectively.

### Definition of the Abrupt State of HR Pathway

We downloaded the gene list of KEGG_HOMOLOGOUS_RECOMBINATION (Supplementary Table S4) from MsigDB database (Liberzon et al., 2011). Firstly, the synonymous mutations in the mutation data from Local-CRC, Mondaca-CRC and TCGA-CRC were deleted, retaining only the mutation data of non-synonymous mutation types. According to the gene list of KEGG_homologus_recombination (HR), we counted the mutation number of the HR pathway in each patient. When the number of gene mutations in a patient’s HR pathway was zero, we labeled it the HR wild-type (WT). Otherwise, they were referred to as the HR mutant-type (MUT).

### Relationship Between HR Pathway Mutation and Prognosis or Curative Effect of CRC Patients

We used logistic regression analysis to explore the relationship between the mutation status of HR pathway and whether Local-CRC patients had metastasis after receiving chemotherapy. In Mondaca-CRC cohort, univariable and multivariate Cox regression analysis, as well as Kaplan-Meier analysis, were used to evaluate the influence of HR pathway mutation on the survival time of CRC patients after receiving chemotherapy.

### Path Enrichment Analysis

Using clusterProfiler R package and gene set enrichment analysis (GSEA) (Yu et al., 2012), we analyzed the expression profile data of the HR-MUT and HR-WT groups and compared the enrichment scores and *p* values of the HR-MUT and HR-WT groups to those of GO-BP, GO-CC, GO-MF, KEGG, and REACTOME. We also used single sample GSEA (ssGSEA) to analyze the pathway score of each CRC patient (Lin et al., 2022).

### Statistical Analysis

The Mann-Whitney U test was used to compare the differences between the continuity variables of the HR-MUT and HR-WT groups, while Fisher’ exact test was used to compare the differences in the classification variables of the HR-MUT and HR-WT groups. The log-rank test in combination with Kaplan-Meier analysis was used to calculate the *p* value. All statistical analysis and visual analysis in this study were based on R language. Here, the *p* value was bilateral and any *p* value of less than 0.05 was regarded as statistically significant.

## Results

### HR-MUT is Related to a Better Curative Effect and Prognosis in CRC Patients Receiving Chemotherapy

**FIGURE 1:**
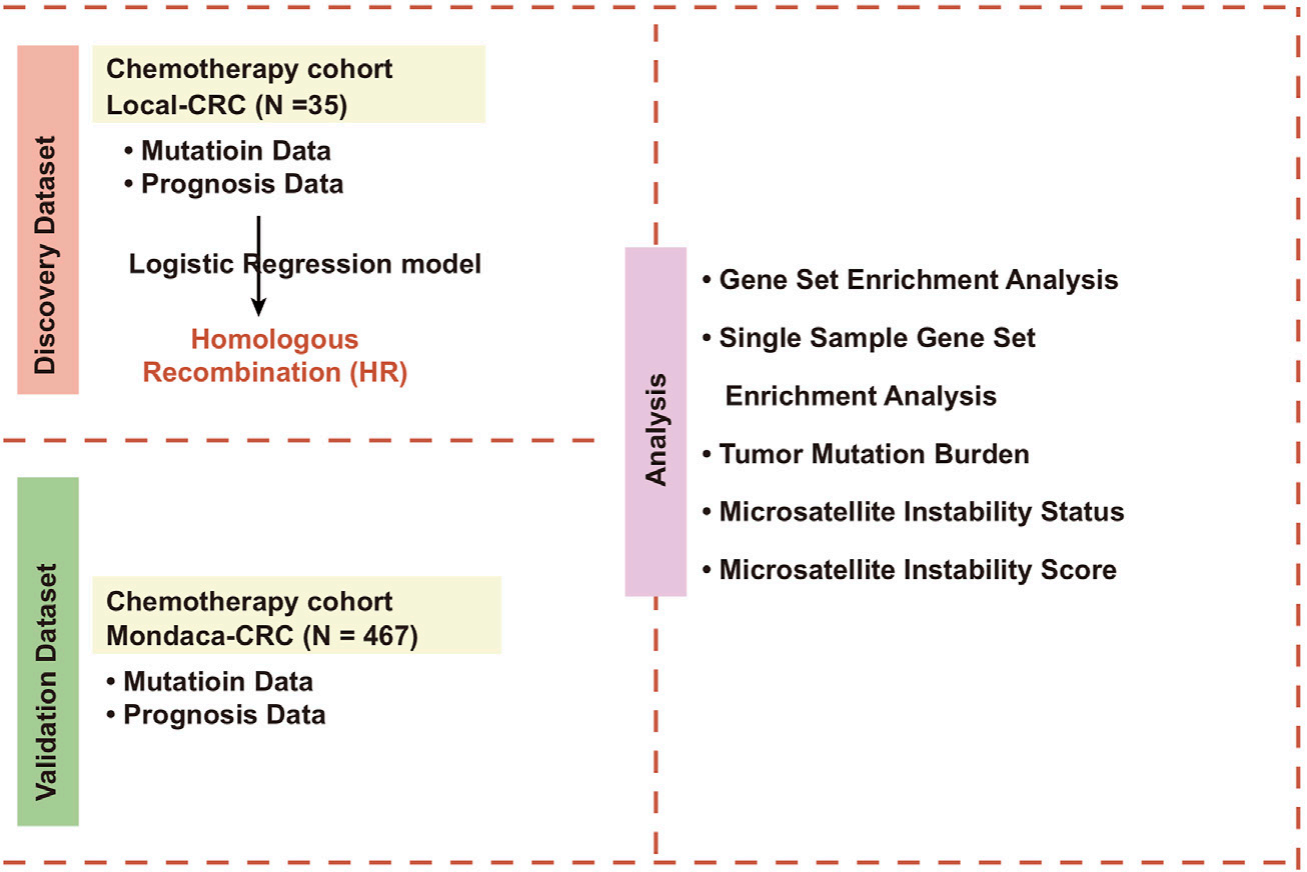
Overall design of the study.

**FIGURE 2:**
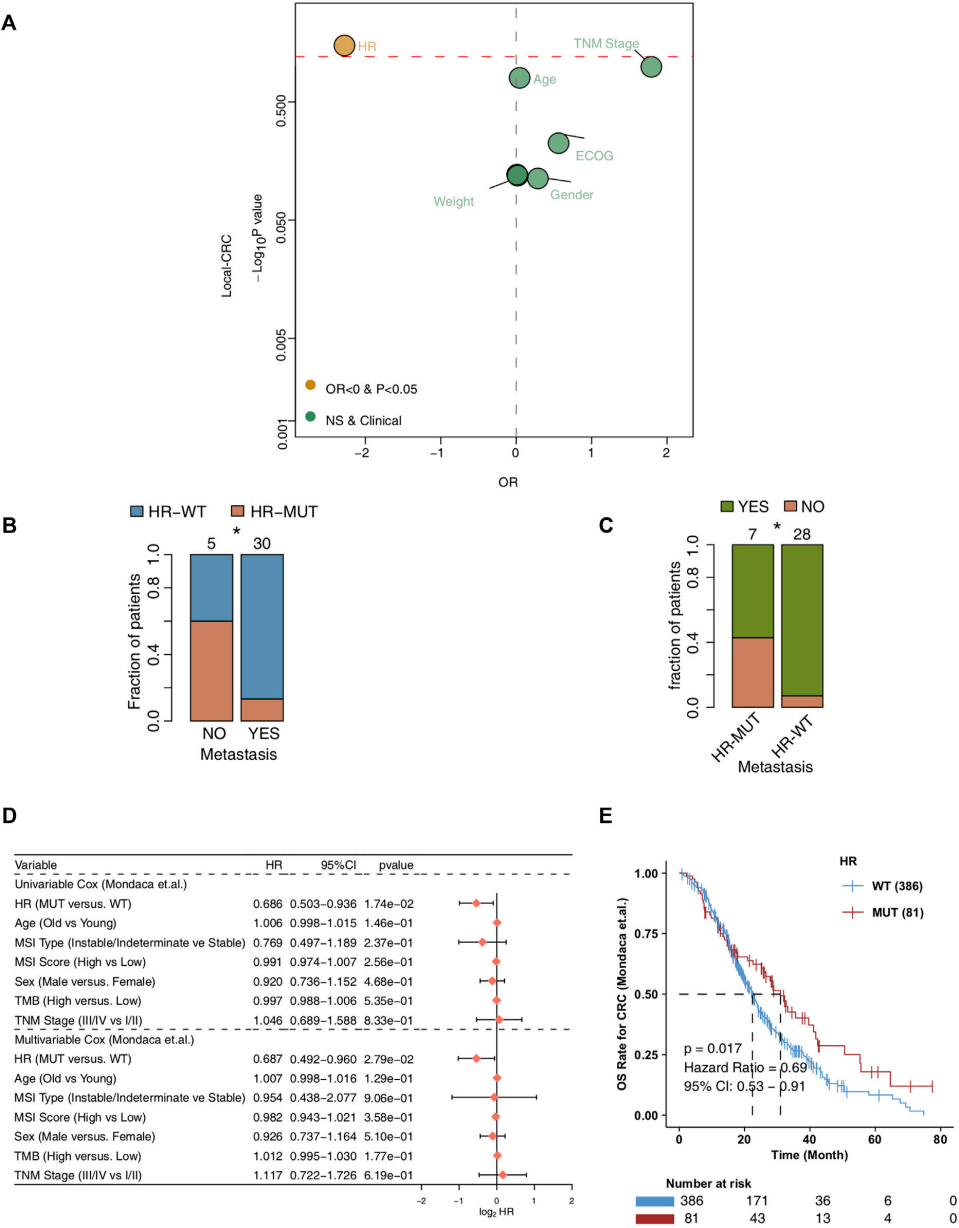
The prognosis value of the HR-MUT group **(A)**. Logistic regression model of the HR-MUT group and clinical characteristics in the Local-CRC cohort **(B)**. A comparison between the HR mutation statuses of the metastasis-yes group and metastasis-no group **(C)**. A comparison between the tumor metastasis statuses of the HR-MUT and HR-WT groups **(D)**. The univariable and multivariable cox regression model of the HR-MUT group and clinical characteristics in the Mondaca-CRC cohort **(E)**. KM curve showed the HR-MUT CRC patients displayed a significant improvement in OS time compared with the HR-WT CRC patients in the Mondaca-CRC cohort (*p < 0.05).

In order to explore the relationship between the mutation state of HR pathway and the chemotherapy efficacy and prognosis of CRC patients (Figure 1), we first analyzed whether HR-MUT can influence the presence of tumor metastasis in CRC patients after chemotherapy (Figure 2A). The results showed that HR-MUT might indeed cause less tumor metastasis in CRC patients after chemotherapy (OR < 0; *p* < 0.05). The results displayed in Figure 2B demonstrate how HR-WT was often found in CRC patients without tumor metastasis after chemotherapy (*p* < 0.05). Meanwhile, as shown in Figure 2C, CRC patients with HR-MUT often did not display any tumor metastasis after receiving chemotherapy (*p* < 0.05). For Mondaca-CRC patients, our univariable and multivariate Cox regression analysis showed that HR-MUT could be used as an independent predictor of chemotherapy prognosis for CRC patients (Figure 2D). Notably, the Kaplan-Meier curve showed that HR-MUT had significantly longer OS time than HR-WT (log-rank *p* = 0.017; HR = 0.69; Figure 2E).

**FIGURE 3:**
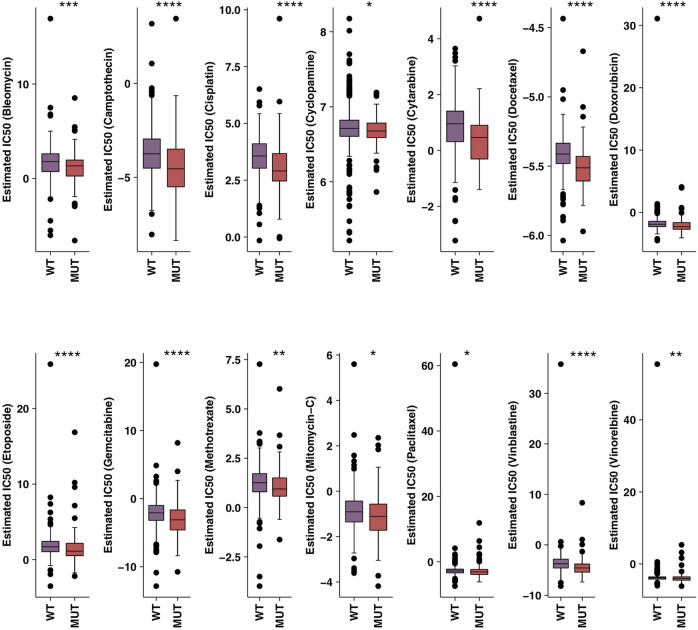
A comparison between the IC50 values of common chemotherapy drugs used on HR-MUT and the HR-WT CRC patients (*p < 0.05; **p < 0.01; ***p < 0.001; ****p < 0.0001).

In order to further verify the relationship between HR-MUT and the sensitivity of chemotherapeutic drugs, we evaluated the IC50 value of several chemotherapeutic drugs using pRRophetic algorithm combined with the CRC patient expression data. From this analysis, we found that HR-MUT group displayed a significantly lower IC50 value with the chemotherapeutic drugs than the HR-WT group (Figure 3).

### HR-MUT is Associated With Higher Mutation Frequency, TMB, and MSIscore

**FIGURE 4:**
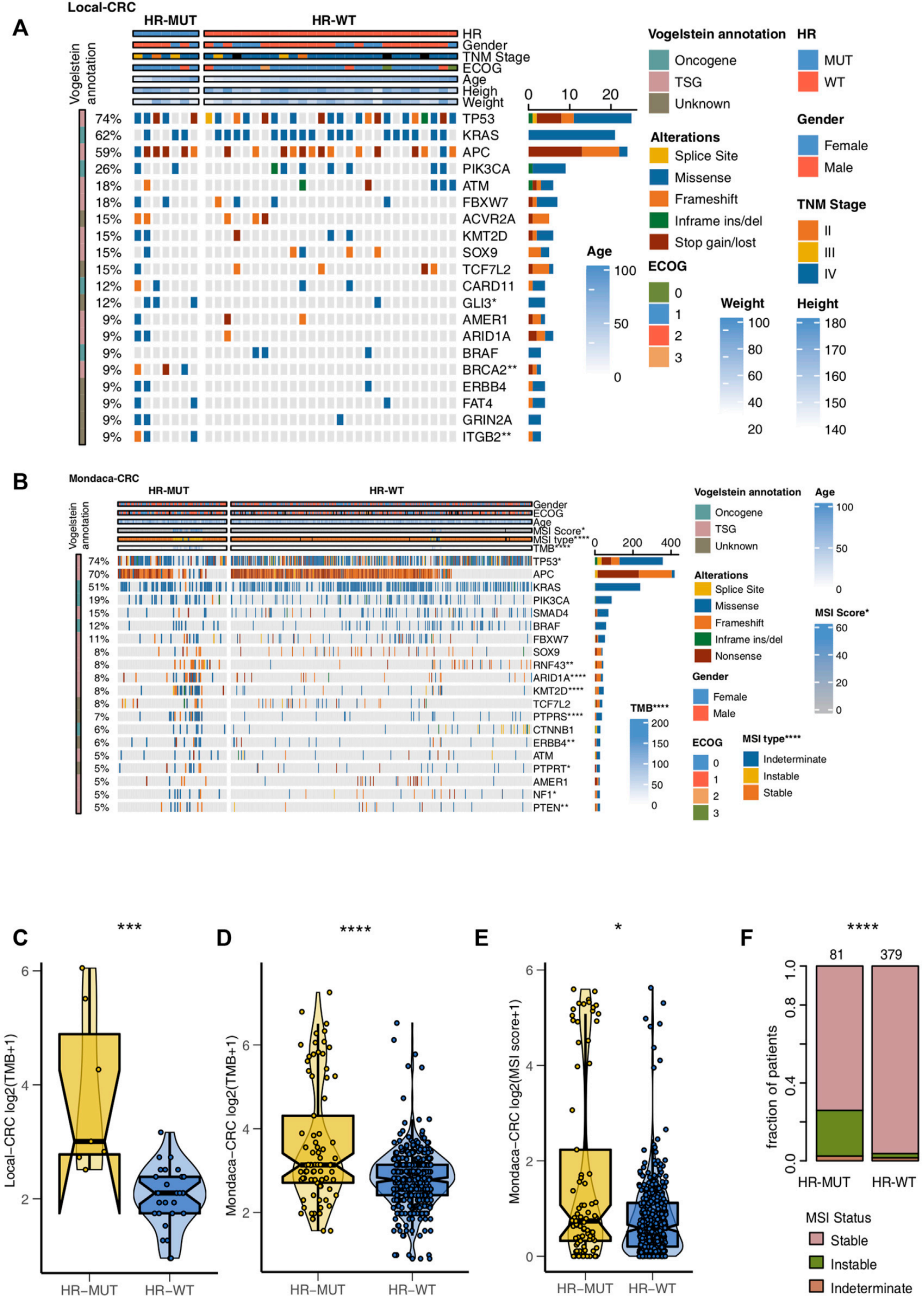
The mutation profiles of the HR-MUT and HR-WT CRC patients. The mutational landscape of the top mutated gene mutations in the Local-CRC cohort **(A)** and Mondaca-CRC cohort **(B)**. A comparison of the TMB levels of the HR-MUT group and HR-WT group in the Local-CRC cohort **(C)** and Mondaca-CRC cohort **(D)**. A comparison between the MSI scores **(E)** and MSI statuses **(F)** the HR-MUT and HR-WT groups in the Mondaca-CRC cohort (*p < 0.05; **p < 0.01; ***p < 0.001; ****p < 0.0001).

Next, we compare the gene mutation frequencies of the HR-MUT and HR-WT groups to the top 20 mutation frequencies found in the other CRC cohorts. For Local-CRC, the HR-MUT group had significantly higher mutation frequency than the HR-WT group in *Gli3* (42.9% vs. 3.6%), *BRCA2* (42.9% vs. 0.0%), and *ITGB2* (42.9% vs. 0.0%) (all *p* < 0.05; Figure 4A). In Mondaca-CRC, the HR-MUT group was notably higher than the HR-WT group in *RNF43* (17.3% vs. 6.2%), *ARID1A* (21.0% vs. 4.9%), *KMT2D* (22.2% vs. 4.7%), *PTPRS* (18.5% vs. 4.7%), *ERBB4* (12.3% vs. 4.1%), *PTPRT* (11.1% vs. 4.1%), *NF1* (9.9% vs. 4.1%), and *PTEN* (11.1% vs. 3.6%), all of which had significantly increased mutation frequency (all *p* < 0.05; Figure 4B). Supplementary Figures S1A,B shows the mutual exclusion and co-occurrence of the top 20 gene mutations in the Local-CRC and Mondaca-CRC cohorts, respectively, while Supplementary Figures S2A,B show the gene mutation of HR-MUT patients in the Local-CRC Cohort and Mondaca-CRC Cohort, respectively.

Compared with HR-WT, HR-MUT displayed a significantly higher level of TMB in Local-CRC and Mondaca-CRC (*p* = 2.55e-04, Figure 4C; P = 5e-05, Figure 4D). We also found that HR-MUT had a higher MSI score than HR-WT (*p* = 2.66e-02; Figure 4E). Similarly, HR-MUT had a higher prevelence of instable MSI status (*p* < 0.0001; Figure 4F).

### HR-MUT is Related to the Less Active DNA Repair Ability of CRC Patients and Promoting Tumor Metastasis and Metabolism

**FIGURE 5:**
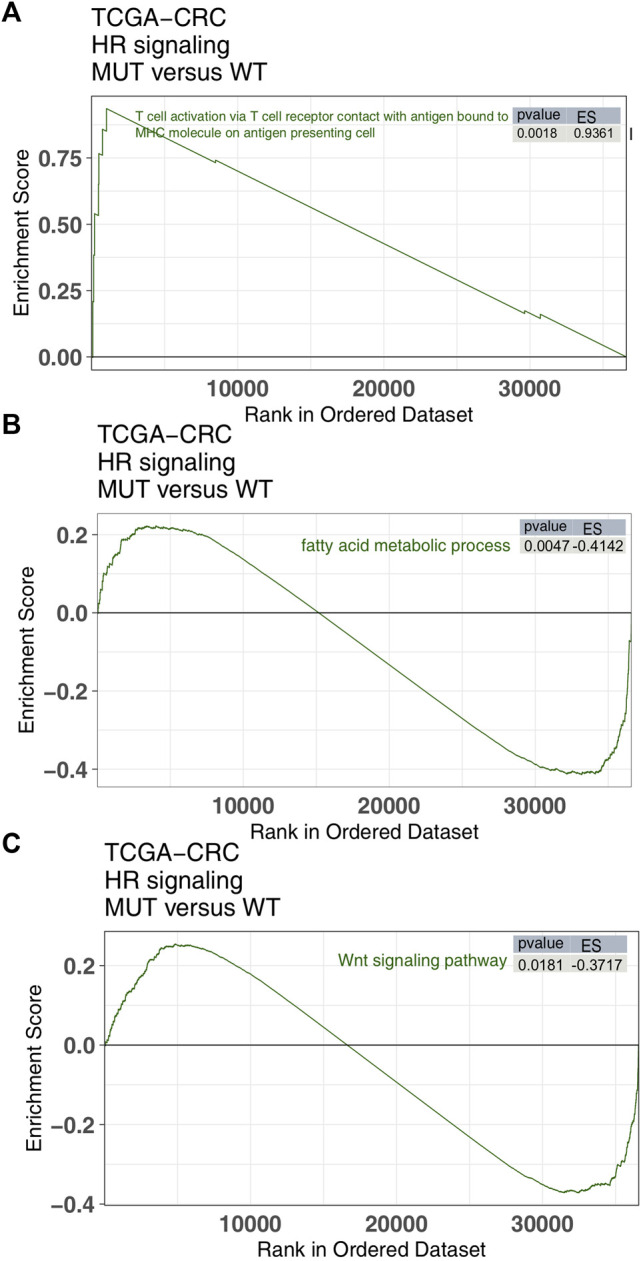
The GSEA results. A comparison of the enrichment scores of immune-related **(A)**, tumor metabolism **(B)** and tumor metastasis/growth **(C)** found in the HR-MUT and HR-WT groups based on the GSEA algorithm.

In order to further explore the difference between HR-MUT and HR-WT in pathway activity, we utilized GSEA and ssGSEA. Compared with HR-WT, HR-MUT displayed a significant increase in immune activity, which is manifested by an increase in antigen presenting ability, infiltration and recruitment of CD4^+^ T cells, and production of INF-gamma (ES > 0, *p* < 0.05, Figure 5A; Supplementary Table S5). Additionally, HR-MUT had significantly reduced tumor metabolic activity, as well as fatty acid metabolic activity (ES < 0, *p* < 0.05; Figure 5B; Supplementary Table S5). Compared with HR-WT, HR-MUT also had significantly reduced WNT pathway activity (ES < 0, *p* < 0.05; Figure 5C; Supplementary Table S5).

**FIGURE 6:**
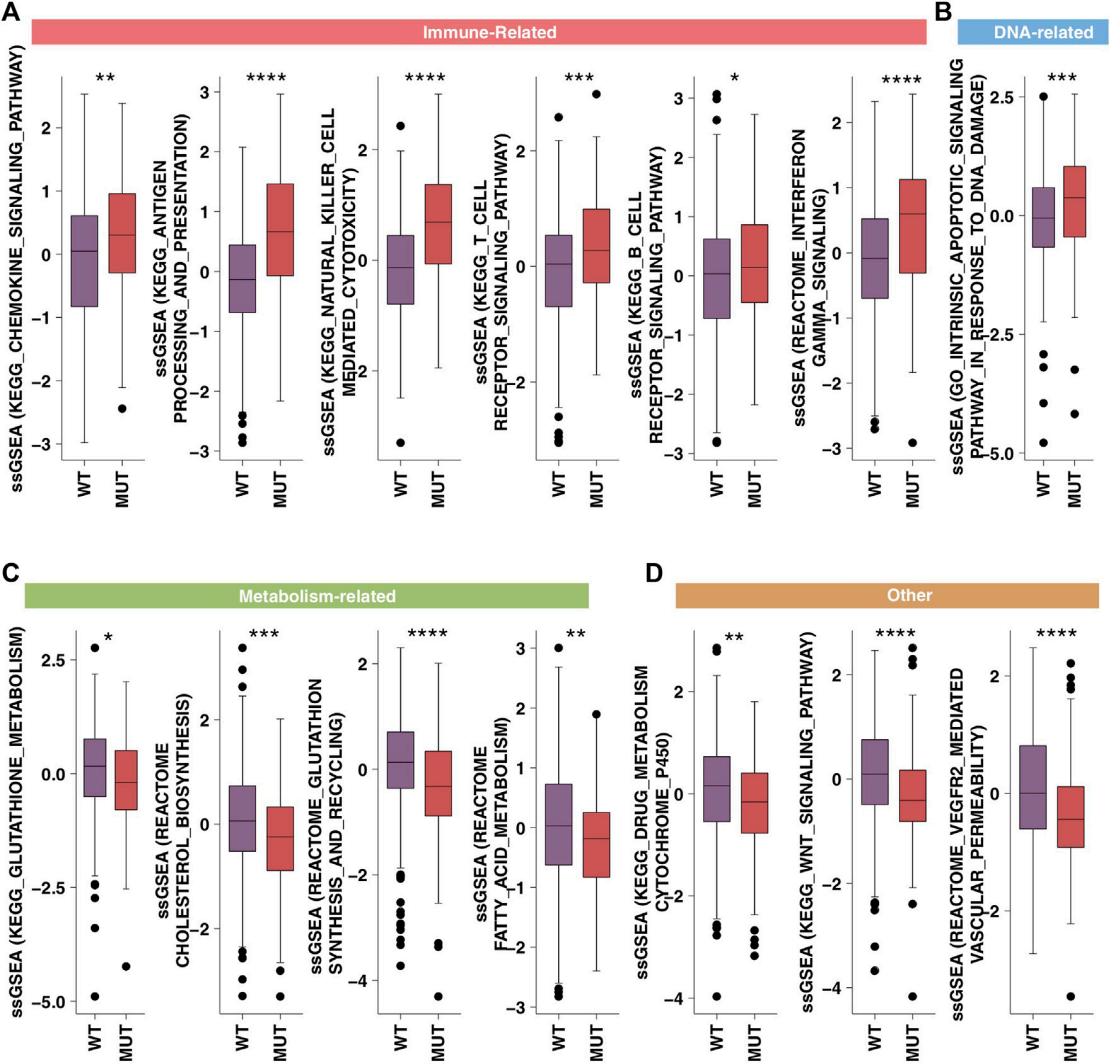
The ssGSEA results. A comparison between the ssGSEA scores in the category of immune-related **(A)**, DNA-related **(B)**, metabolism-related **(C)** and tumor metastasis/growth related **(D)** of the HR-MUT and HR-WT based on the ssGSEA algorithm (*p < 0.05; **p < 0.01; ***p < 0.001; ****p < 0.0001).

In order to further verify the channel activity, we used the ssGSEA algorithm and obtained similar results (Figures 6A–D). From this, we found that HR-MUT had significantly higher immune response ability than HR-WT (Figure 6A), while HR-MUT had significantly lower DNA repair ability, tumor metabolism ability, and tumor metastasis ability (Figures 6B–D).

## Discussion

In this study, we found that HR-MUT is related to the reduced occurrence of tumor metastasis in CRC patients who have received chemotherapy. Additionally, HR-MUT was related to a significantly longer OS time than HR-WT for CRC patients receiving chemotherapy. More importantly, univariate and multivariate cox regression models showed that HR-MUT acts as an independent prognostic marker for CRC patients receiving chemotherapy. From the data derived from the genome level analysis, we found that HR-MUT CRC patients had significantly increased TMB and MSI scores, as well as MSI-Instable status, in comparison to HR-WT CRC patients. Based on the pathway enrichment analysis, HR-MUT patients displayed a significant reduction in their DNA repair ability, tumor survival or metastasis related activity, and tumor metabolic activity. Conversely, HR-MUT patients displayed a much higher level of immune response activity. We also found that HR-MUT CRC patients had a longer survival time after receiving immunotherapy than HR-WT CRC patients. Therefore, we speculate that HR-MUT may be a valuable prognostic marker for CRC patients receiving chemotherapy.

The stronger immune response in the tumor microenvironment may be one of the potential mechanisms behind the better prognosis found in HR-MUT patients receiving chemotherapy. In the HR-MUT group, the activity of the interferon gamma production pathway was significantly higher than that of the HR-WT group. In this vein, studies have shown that although chemotherapeutic drugs can directly clear divided endothelial cells by inhibiting enzymes involved in DNA replication or microtubule metabolism, most of the vascular injuries may be caused by chemotherapy-induced inflammation (Kerbel and Kamen, 2004; Lee and Wu, 2015). This is due to the fact that in the process of tumor rejection induced by cyclopamine, immune downregulation is activated to produce interferon-γ (IFN-γ), thus inhibiting angiogenesis (Ibe et al., 2001). IFNγ-γ-mediated vascular inhibition is one of the important anti-tumor mechanisms of T cells (Qin and Blankenstein, 2000; Kammertoens et al., 2017), as it can reduce the expression of Dll4 signaling pathway in endothelial cells, destroy the connection between endothelial cells, and resist angiogenesis in tumor tissue (Deng et al., 2014). However, in a study on early treatment of tumors that combined IFNγ with chemotherapy, it was found that IFNγ could in fact increase the mortality rate (Alberts et al., 2008; Zarogoulidis et al., 2013). As for the activity of IL-6 production, the rates in HR-MUT were significantly lower than that in HR-WT. Further research on this topic has revealed that IL-6 activates the STAT3 signaling pathway of tumor cells and promotes the expression of VEGF, thereby inducing angiogenesis (Wei et al., 2003). We also found that the activation of macrophages in the HR-MUT group was significantly lower than that in the HR-WT group. Earlier research on this shows that macrophages are one of the main cell types in the tumor microenvironment, and they can promote cell survival and chemotherapy resistance by releasing interleukin -17 (Guo et al., 2017). Similarly, according to an earlier study by Ruffell. et al., it was found that M2 macrophage inhibited the production of IL-12 by dendritic cells through the over-secretion of IL-10, therefore blocking the immune response of CD8+T cells (Ruffell et al., 2014).

Tumor metabolic reprogramming may also be one of the important mechanisms involved in CRC chemotherapy resistance. In our study, we found that lipid metabolism was significantly down-regulated in HR-MUT. Beyond our own results, other studies have confirmed that lipid metabolism rearrangement is involved in the regulation of the CRC cell chemotherapy resistance mechanism (Fiucci et al., 2000; Cotte et al., 2018), plays a role in the occurrence and development of CRC (Hwang et al., 2014; Liu et al., 2016; Mihajlovic et al., 2019; Liu et al., 2020), and has an impact on the prognosis (de Figueiredo Junior et al., 2018). Research has also found that lipid droplets (LD) levels in CRC cells are significantly higher than in those of normal cells, indicating that it may play a key role in the development of CRC (Accioly et al., 2008). Many studies have confirmed that lipid metabolism-related enzymes participate in the occurrence and development of CRC (Liu et al., 2020), regulate cell proliferation and invasion (Sánchez-Martínez et al., 2015; Nath and Chan, 2016), and participate in drug resistance (Fiucci et al., 2000; Cotte et al., 2018). Because of this, fatty acids, phospholipids, and cholesterol are synthesized actively, and their concentrations are significantly increased in tumor tissues (Janardhan et al., 2006; Lupu and Menendez, 2006). With this in mind, some anti-tumor drugs have been developed to significantly reduce cell cholesterol levels by blocking different steps of cholesterol biosynthesis (Eliassen et al., 2005; Gorin et al., 2012; Luu et al., 2013). Except for lipid metabolism, we found that the activity of glutathione synthesis in HR-MUT group was significantly lower than that in HR-WT group. Glutamine metabolism can also promote drug resistance in tumors by influencing the post-translational modification of proteins. Treatment with glutamine analogue DON can increase the sensitivity of pancreatic cancer to gemcitabine. In past studies, researchers have found that DON can inhibit hexosamine pathways, which leads to a change in the glycosylation level of protein groups in the cell as while. This ultimately affects the sensitivity of tumors to chemotherapy (Chen et al., 2017). Additionally, glutamine metabolism can affect the drug resistance of tumors epigenetically. In *KRAS* mutant colon cancer cells, *SLC25A22* promotes the accumulation of succinic acid, a metabolite of glutamine in the tricarboxylic acid cycle, which further increases the local methylation degree of DNA and then promotes the activation of Wnt signaling pathway. This sequence of events leads to tolerance of colon cancer cells to chemotherapeutic drugs (Wong et al., 2020).

In addition to immune response and tumor metabolism reprogramming, pathways related to tumor growth and metastasis may also be involved in CRC chemotherapy resistance. Based on GSEA and ssGSEA, we found that the WNT pathway activity was significantly upregulated in HR-WT. According to Nagaraj et al., the expression levels of β-catenin and survivin in A549 sensitive and drug-resistant cells were higher than those in sensitive cells after cisplatin treatment (Nagaraj et al., 2015). Furthermore, Novetsky et al.‘s research indicated that inhibiting the activity of GSK-3β in cells can activate the WNT pathway, which mediates chemotherapy resistance (Novetsky et al., 2013). Zhang et al. found that interfering with β-catenin using siRNA can improve the sensitivity of tumor cells to chemotherapeutic drugs, further proving that the WNT pathway plays an important role in chemotherapeutic drug resistance (Zhang et al., 2015). In our own research, we found that VEGFR2 pathway was significantly activated in HR-WT. Similarly, Mann et al. inhibited the phosphorylation of VEGFR2 in tumor cells and observed that while blood vessels in the tumor remained normal, the sensitivity of the tumor cells to chemotherapeutic drugs such as cyclophosphamide and cisplatin was enhanced (Stockmann et al., 2008).

## Conclusion

In summary, our research shows that HR-MUT is associated with less tumor metastasis in CRC patients after receiving chemotherapy. The HR-MUT CRC patients had significantly improved OS time and, as such, HR-MUT could be used as a predictor of chemotherapy prognosis in the future treatment plan. Additionally, HR-MUT patients had higher TMB levels, MSI scores, and immune response activity, while they also had lower tumor survival, metastasis pathway activity, and tumor metabolic reprogramming activity. The activity of these pathways may greatly increase the sensitivity of HR-MUT patients to chemotherapy drugs. Therefore, HR-MUT may be used as a new predictive marker for CRC patients who are receiving chemotherapy treatment.

## Data Availability

The original contributions presented in the study are included in the article/Supplementary Material, further inquiries can be directed to the corresponding authors.

## Supplementary Material

Supplementary Figure S1 The co-occurrence and mutually exclusive occurrence of the top 20 mutated genes in the Local-CRC **(A)** and the Mondaca-CRC **(B)** cohorts.

Supplementary Figure S2 The mutation landscape of the HR mutations of the HR-MUT CRC patients in the Local-CRC **(A)** and the Mondaca-CRC **(B)** cohorts.

Supplementary Table S1 The clinical characteristics of the Local-CRC cohort.

Supplementary Table S2 The clinical characteristics of the Mondaca-CRC cohort.

Supplementary Table S3 The clinical characteristics of the TCGA-CRC cohort.

Supplementary Table S4 The gene set of the HR signaling used in this study.

Supplementary Table S5 The GSEA result of the enrichment scores between the HR-MUT and HR-WT groups based on the GSEA algorithm.

## Abbreviation

CRC, colorectal cancer; ECOG, Eastern Cooperative Oncology Group Performance Status; GSEA, gene set enrichment analysis; HR, Homologous recombination; HR-MUT, HR mutant-type; HR-WT, HR wild-type; IC50, half maximal inhibitory concentration; HRD, homologous recombination defect; LD, lipid droplets; IFN-γ, interferon-γ; MSI: Microsatellite instability; NER, Nucleotide excision repair; OS, overall survival; TMB, Tumor mutational burden, ssGSEA, single sample gene set enrichment analysis.
