# Supplementary material for: Homologous Recombination Pathway Alternation Predicts Prognosis of Colorectal Cancer With Chemotherapy
Source: Front Pharmacol. 2022 Jun 6;13:920939. doi: 10.3389/fphar.2022.920939 (PMC9207269; doi:10.3389/fphar.2022.920939)
Supplement: Supplementary file 1 [file DataSheet2.PDF]

	HR-MUT (N=81)	HR-WT (N=386)	Overall (N=467)	P-Value
**Gender**				
Female	34 (42.0%)	198 (51.3%)	232 (49.7%)	**P > 0.05**
Male	47 (58.0%)	188 (48.7%)	235 (50.3%)	
**TNMStage**				
I	1 (1.2%)	7 (1.8%)	8 (1.7%)	
II	8 (9.9%)	23 (6.0%)	31 (6.6%)	
III	18 (22.2%)	71 (18.4%)	89 (19.1%)	**P > 0.05**
IV	54 (66.7%)	285 (73.8%)	339 (72.6%)	
**ECOG**				
0	35 (43.2%)	151 (39.1%)	186 (39.8%)	
1	38 (46.9%)	166 (43.0%)	204 (43.7%)	**P > 0.05**
2	5 (6.2%)	26 (6.7%)	31 (6.6%)	
3	0 (0%)	1 (0.3%)	1 (0.2%)	
Missing	3 (3.7%)	42 (10.9%)	45 (9.6%)	
**MSI TYPE**				
Indeterminate	2 (2.5%)	6 (1.6%)	8 (1.7%)	
Instable	19 (23.5%)	8 (2.1%)	27 (5.8%)	
Stable	60 (74.1%)	365 (94.6%)	425 (91.0%)	**P < 0.05**
Missing	0 (0%)	7 (1.8%)	7 (1.5%)	
**TMB**				
Mean (SD)	20.5 (28.1)	7.10 (7.07)	9.43 (14.2)	**P < 0.05**
Median [Min, Max]	7.78 [1.96, 152]	5.87 [0.865, 91.0]	6.05 [0.865, 152]	
**Age**				
Mean (SD)	60.9 (14.4)	58.8 (13.1)	59.2 (13.4)	**P > 0.05**
Median [Min, Max]	60.0 [26.0, 95.0]	59.0 [18.0, 93.0]	59.0 [18.0, 95.0]	
**MSI SCORE**				
Mean (SD)	8.21 (14.3)	1.31 (4.26)	2.51 (7.55)	
Median [Min, Max]	0.670 [0, 47.4]	0.500 [-1.00, 48.5]	0.550 [-1.00, 48.5]	**P < 0.05**
Missing	0 (0%)	1 (0.3%)	1 (0.2%)	
