# Supplementary figures and images for: Homologous Recombination Pathway Alternation Predicts Prognosis of Colorectal Cancer With Chemotherapy
Source: Front Pharmacol. 2022 Jun 6;13:920939. doi: 10.3389/fphar.2022.920939 (PMC9207269; doi:10.3389/fphar.2022.920939)

**A**

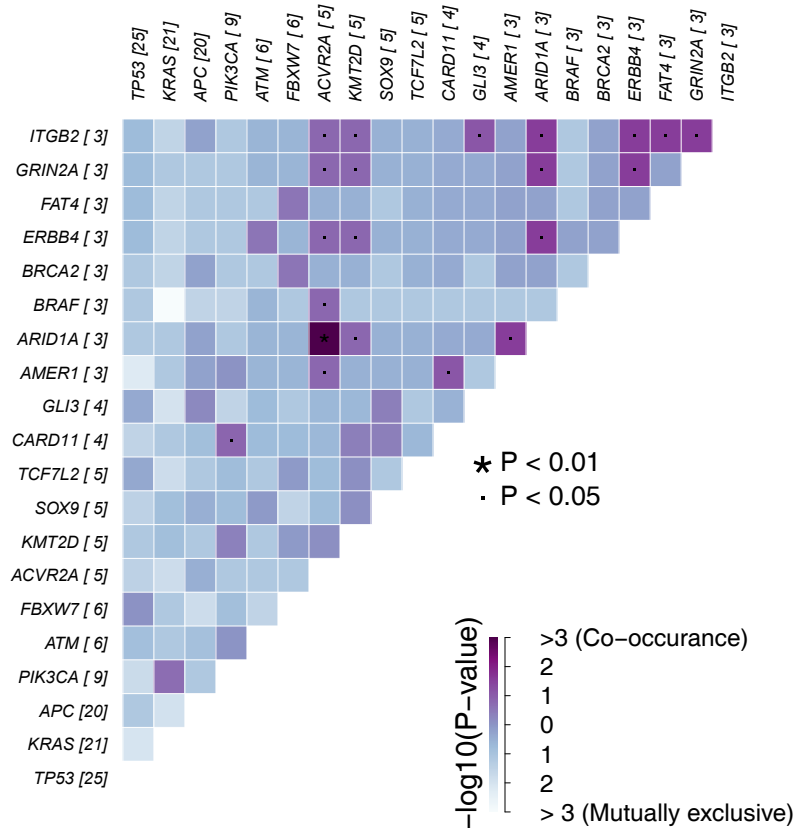

**B**

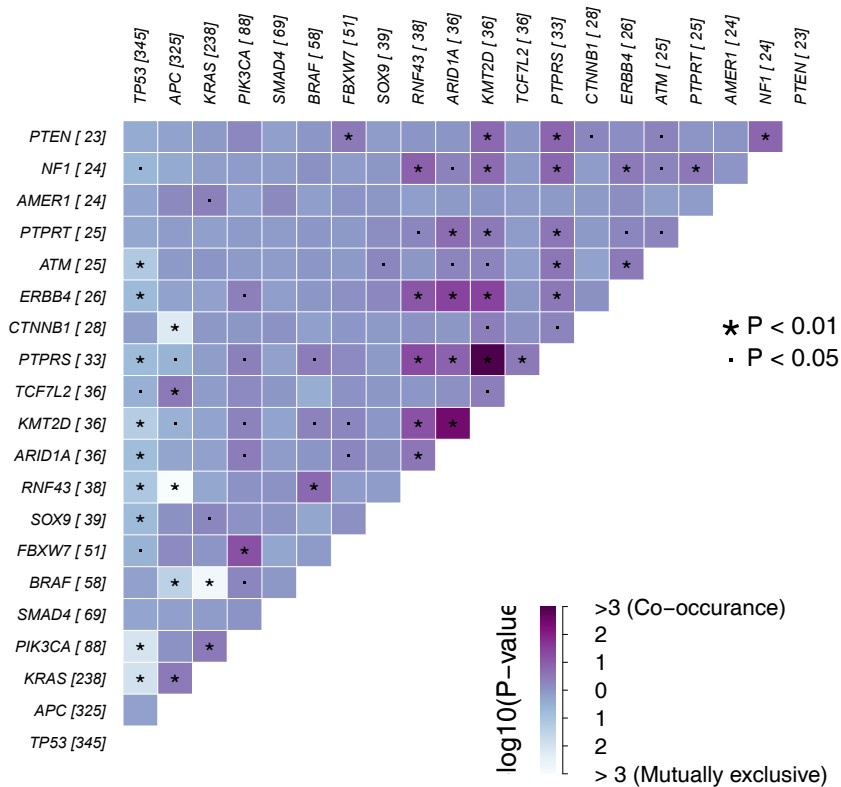

Supplement: Supplementary file 3 [file Image2.PDF]

A

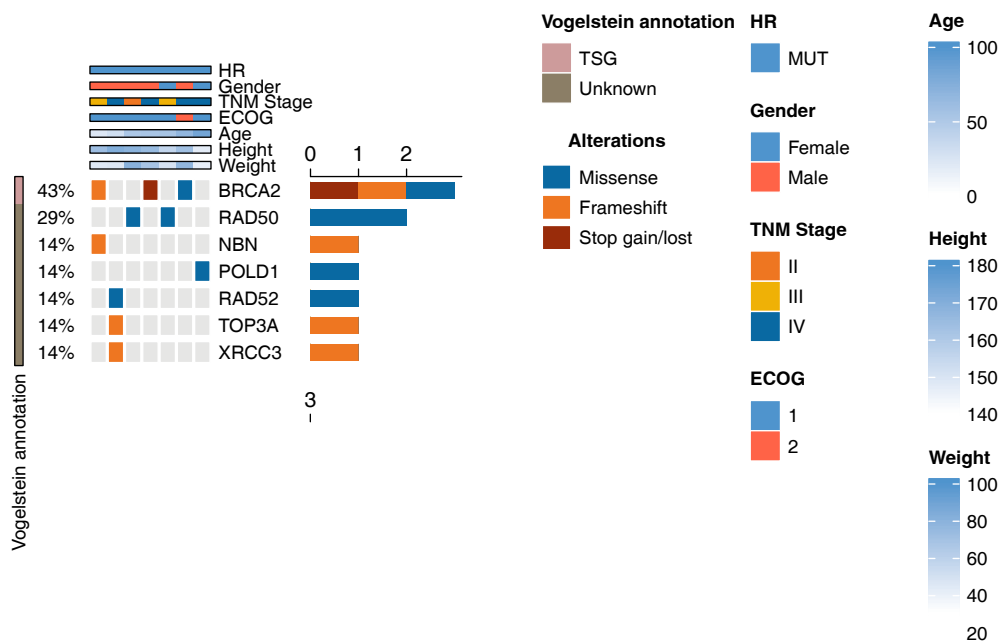

B

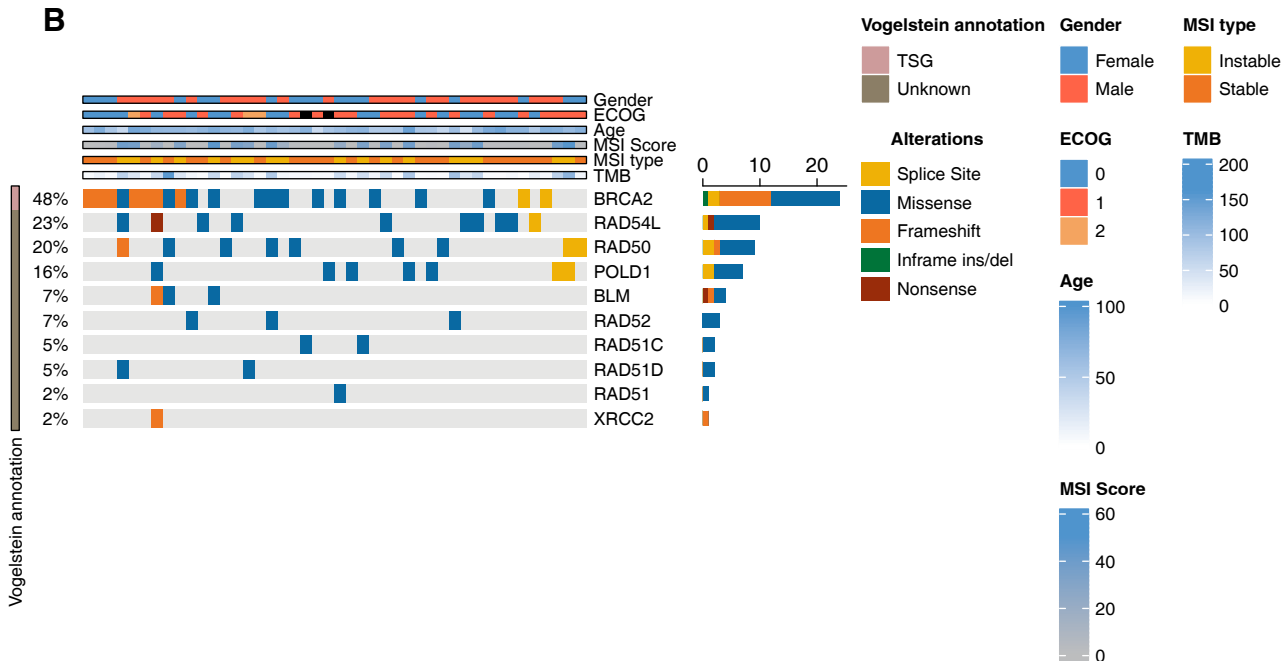

Supplement: Supplementary file 7 [file Image1.PDF]
