# Supplementary material for: Homologous Recombination Pathway Alternation Predicts Prognosis of Colorectal Cancer With Chemotherapy
Source: Front Pharmacol. 2022 Jun 6;13:920939. doi: 10.3389/fphar.2022.920939 (PMC9207269; doi:10.3389/fphar.2022.920939)
Supplement: Supplementary file 4 [file DataSheet3.PDF]

	HR-MUT (N=116)	HR-WT (N=413)	Overall (N=529)	PValue
**Gender**				
female	62 (53.4%)	188 (45.5%)	250 (47.3%)	
male	54 (46.6%)	222 (53.8%)	276 (52.2%)	**P > 0.05**
Missing	0 (0%)	3 (0.7%)	3 (0.6%)	
**TNM Stage**				
I	20 (17.2%)	68 (16.5%)	88 (16.6%)	
II	56 (48.3%)	134 (32.4%)	190 (35.9%)	
III	31 (26.7%)	121 (29.3%)	152 (28.7%)	**P < 0.05**
IV	7 (6.0%)	69 (16.7%)	76 (14.4%)	
Missing	2 (1.7%)	21 (5.1%)	23 (4.3%)	
**Ethnicity**				
hispanic or latino	1 (0.9%)	4 (1.0%)	5 (0.9%)	
not hispanic or latino	71 (61.2%)	261 (63.2%)	332 (62.8%)	**P > 0.05**
Missing	44 (37.9%)	148 (35.8%)	192 (36.3%)	
**Age**				
Mean (SD)	66.6 (14.3)	66.0 (12.4)	66.1 (12.8)	
Median [Min, Max]	67.0 [33.0, 90.0]	68.0 [31.0, 90.0]	68.0 [31.0, 90.0]	**P > 0.05**
Missing	0 (0%)	3 (0.7%)	3 (0.6%)	
