# Supplementary material for: Homologous Recombination Pathway Alternation Predicts Prognosis of Colorectal Cancer With Chemotherapy
Source: Front Pharmacol. 2022 Jun 6;13:920939. doi: 10.3389/fphar.2022.920939 (PMC9207269; doi:10.3389/fphar.2022.920939)
Supplement: Supplementary file 5 [file DataSheet1.PDF]

	HR-MT (N=7)	HR-WT (N=28)	Overall (N=35)	P-Value
**Metastasis**				
NO	3 (42.9%)	2 (7.1%)	5 (14.3%)	**P < 0.05**
YES	4 (57.1%)	26 (92.9%)	30 (85.7%)	
**Gender**				
Female	2 (28.6%)	10 (35.7%)	12 (34.3%)	**P > 0.05**
Male	5 (71.4%)	18 (64.3%)	23 (65.7%)	
**TNMStge**				
II	1 (14.3%)	1 (3.6%)	2 (5.7%)	
III	2 (28.6%)	2 (7.1%)	4 (11.4%)	**P > 0.05**
IV	4 (57.1%)	22 (78.6%)	26 (74.3%)	
Missing	0 (0%)	3 (10.7%)	3 (8.6%)	
**ECOG**				
0	0 (0%)	2 (7.1%)	2 (5.7%)	
1	6 (85.7%)	22 (78.6%)	28 (80.0%)	**P > 0.05**
2	1 (14.3%)	3 (10.7%)	4 (11.4%)	
3	0 (0%)	1 (3.6%)	1 (2.9%)	
**Age**				
Mean (SD)	51.3 (20.9)	49.7 (16.0)	50.0 (16.8)	**P > 0.05**
Median [Min, Max]	53.0 [22.0, 83.0]	50.0 [14.0, 84.0]	51.0 [14.0, 84.0]	
**Height**				
Mean (SD)	162 (8.04)	165 (7.49)	164 (7.57)	**P > 0.05**
Median [Min, Max]	164 [147, 170]	165 [148, 176]	165 [147, 176]	
**Weight**				
Mean (SD)	57.2 (16.7)	60.5 (12.9)	59.8 (13.5)	**P > 0.05**
Median [Min, Max]	48.0 [40.5, 80.0]	63.3 [38.0, 87.0]	62.5 [38.0, 87.0]	
